# Adjustment for Inconsistency in Adaptive Phase 2/3 Designs With Dose Optimization

**DOI:** 10.1002/pst.70031

**Published:** 2025-09-26

**Authors:** Cong Chen, Mo Huang, Xuekui Zhang

**Affiliations:** ^1^ Biostatistics and Research Decision Sciences, Merck & Co., Inc Rahway New Jersey USA; ^2^ Pfizer Inc Collegeville Pennsylvania USA; ^3^ Department of Mathematics and Statistics University of Victoria Victoria British Columbia Canada

**Keywords:** adaptive design, dose optimization, inconsistency, phase 2/3 trial, seamless design, type I error control

## Abstract

Adaptive Phase 2/3 designs hold great promise in contemporary oncology drug development, especially when limited data from Phase 1 dose‐finding is insufficient for identifying an optimal dose. However, inconsistent results between Phase 2 and Phase 3 may raise regulatory and practical concerns. The imperfection in dose selection further complicates the issue. In this paper, we explicitly incorporate the concerns about inconsistency into the statistical analysis under three hypothesis testing strategies (conservative, aggressive, and neutral) by specifying an inconsistency cutoff and accounting for the probability of “picking‐the‐winner.” This investigation illustrates how to balance regulatory caution, sponsor interests, and practical considerations in adaptive Phase 2/3 designs with dose optimization, which paves the way for further research in a less explored area.

## 1 Introduction

Adaptive Phase 2/3 designs (sometimes called “seamless” designs) can accelerate oncology drug development by using Phase 2 data to decide whether, and how, to proceed into Phase 3. In practice, however, it is well known that an adaptive Phase 2/3 trial may produce numerically inconsistent results between Stage 1 (Phase 2) patients and Stage 2 (Phase 3) patients, even when there is no change in the standard of care or eligibility criteria between the two stages [1]. This inconsistency could arise because the trial begins with a limited number of sites in a few countries, which may not be representative of all sites used once recruitment is fully underway. It could occur when the expansion from Phase 2 to Phase 3 is perceived as a positive signal, attracting patients with poor prognoses who normally would not participate in any clinical trial, with unpredictable consequences. It could also be due to the difference in follow‐up time between Stage 1 and Stage 2 (e.g., a delayed effect may favor Stage 1 while a crossover effect may favor Stage 2), or simply due to randomness. When inconsistency is detected, it can become a serious regulatory review issue, and the ability to combine results across independent stages to assess the overall treatment effect becomes questionable [2].

An additional layer of complexity arises when Stage 1 includes multiple experimental doses, and a single dose is “picked” for Stage 2. This dose decision is typically based on early safety, tolerability, or surrogate efficacy endpoints. In oncology, dose optimization has gained particular attention through the FDA's Project Optimus. If the early endpoints used to pick the dose correlate imperfectly with the primary endpoint, there is a risk of choosing a suboptimal or “loser” dose, which in turn influences the observed effect size. Most existing adaptive designs assume with certainty that a “winning” dose is carried forward from Phase 2 to Phase 3 [3], hence ignoring the possibility of imperfection in dose selection.

In this paper, we propose a statistical framework that explicitly accounts for potential inconsistency between the two stages and for imperfect dose selection. Specifically, we consider three hypothesis‐testing strategies—conservative, aggressive, and neutral—differing in criterion for including Stage 1 data in the primary analysis based on the observed difference between the two stages. We incorporate a parameter w that represents the true probability that the chosen dose is a genuine “winner,” and a cutoff c that defines the maximum allowable observed difference between Stage 1 and Stage 2 for the data to be pooled. We then derive an adjusted threshold α* from a closed‐form equation to ensure strong control of the overall Type I error at level α.

In what follows, Section 2 describes the methods in detail, including the definition of w, the three testing strategies, and the derivation of α*. Section 3 presents numerical results and figures illustrating how design parameters affect α*. Section 4 discusses the practical implications and trade‐offs among the different strategies. Section 4 concludes. Appendix 1 provides more technical derivations and sample R code, and Appendix 2 provides an upper bound for w.

## 2 Methods

### 2.1 Illustration of Inconsistency in a Simple Two‐Stage Design

To illustrate the concern of inconsistency, consider a simple two‐stage adaptive Phase 2/3 design without dose selection. The primary objective of Stage 1 is to demonstrate drug activity using a surrogate endpoint to make a Go‐No Go decision for expansion to Stage 2. Once demonstrated (e.g., response rate improvement > 10%), the study proceeds to Stage 2 to enroll more patients. The overall study is designed to have 90% power (approximately 510 events) to detect a hazard ratio (HR) of 0.75 in overall survival between an experimental arm and a control arm (1:1 randomization ratio) at the 0.025 one‐sided significance level. The full trial pooled analysis (primary analysis) is performed after complete follow‐up of both stages whereas Stage 1 patients are assumed to contribute 30% of the overall survival information.

Table 1 presents a scenario where the observed HR is approximately 0.84 (significant at the 0.025 level). However, the disparity between Stage 1 and Stage 2 HR estimates can fluctuate notably due to random variability or practical factors. Taking advantage of the approximate relationship between test statistics and $-\log\left(\mathrm{HR}\right)$ (see more details in Section 2.3), a simple calculation indicates that, purely due to randomness, the probability of the absolute difference in the log‐hazard ratio between the two stages exceeding $\log\left(1.1\right)$ or $\log\left(1.2\right)$ is approximately 62% and 35%, respectively.

**TABLE 1:** Survival outcome of a hypothetical adaptive Phase 2/3 trial with Stage 1 contributing 30% of the information, where the overall treatment effect is positive at the 0.025 level based on 510 events (90% power for detecting a hazard ratio of 0.75).

Hazard ratio (overall)	Hazard ratio (Stage 1)	Hazard ratio (Stage 2)	$-\log\left(\mathrm{HR}\right)$ (Stage 1/2)	Nominal *p* (Stage 2)
0.84	0.954	0.795	$-\log\left(1.2\right)$	0.015
0.84	0.898	0.816	$-\log\left(1.1\right)$	0.027
0.84	0.786	0.864	$\log\left(1.1\right)$	0.084
0.84	0.739	0.887	$\log\left(1.2\right)$	0.129

Regulatory agencies are typically concerned when the overall treatment effect is driven by the Stage 1 patients (row 4), while a clearly better outcome in Stage 2 (row 1) may concern both trial sponsors and regulatory agencies.

### 2.2 Imperfect Dose Selection

Building on the previous example, we now consider a scenario where Phase 2 involves selecting between two doses. When the primary efficacy endpoint can be observed immediately, the dose with superior efficacy (i.e., pick‐the‐winner) is carried from Stage 1 to Stage 2 with certainty, a fundamental assumption under prevailing statistical approaches such as the *p* value combination test [3]. However, when the primary endpoint used for dose selection is based on incomplete follow‐up, when the early efficacy endpoint used for dose selection has an uncertain correlation with the primary endpoint [4], or when safety and tolerability are prioritized over efficacy in dose optimization [5, 6], it is unclear whether the selected dose still has superior efficacy. Guided by FDA's Project Optimus, dose selection in oncology is often based on early efficacy and safety data. Imperfection in the dose decision further complicates the inconsistency issue.

Let $w\in \left[0,1\right]$ denote the true probability of picking‐the‐winner under the null hypothesis of no survival difference between the two doses. It depends on the dose selection criteria and the correlations of the endpoints involved in dose selection. Its estimation is outside the scope of this paper, but relevant discussions can be found in [6]. When 0.5<w≤1, Stage 1 patients treated at the selected dose tend to exhibit numerically greater survival benefit than Stage 2 patients treated at the same dose (a “random high”); conversely, when 0≤w<0.5, a “random low” may be observed. While random high may inflate the treatment effect (e.g., rows 3 and 4 in Table 1), a reason why a statistical penalty is paid for Type I error control, random low may deflate the treatment effect (e.g., rows 1 and 2 in Table 1). Let t be the information fraction of the survival data for the selected dose and control contributed by Stage 1 patients. The difference in the observed treatment effect ($-\log\left(\mathrm{HR}\right)$) between Stage 1 and Stage 2 is examined for consistency via a cutoff c (e.g., $\log\left(1.1\right)$ or $\log\left(1.2\right)$ as in Table 1). We focus on the impact of w, c, and t on Type I error control in this paper.

### 2.3 Derivation of the Adjusted (α*) for Overall Type I Error Control

In the adaptive Phase 2/3 design, let N be the overall number of events between the selected dose and the control, and let I=N/4 be the information unit. Suppose there are two experimental doses (j=1,2) in Stage 1, one of which will be selected for Stage 2. Let $Y_{1j}$ be the log‐rank test statistic of overall survival at the end of study in Stage 1 patients at dose level j, and let $Y_{1s}$ be the corresponding log‐rank test statistics for the selected dose. By definition, the probability of picking the “winner”, w, is

$$w\;=\;\mathrm{Pr}\left(Y_{1s}=\max\left\{Y_{11},Y_{12}\right\}\right)$$

Let $Y_{2s}$ be the log‐rank test statistic of overall survival for the selected dose in Stage 2. When w>0.5, $Y_{1s}$ has an increased chance of being a ‘random high’, potentially inflating Type I error if Stage 1 and Stage 2 are combined. When w<0.5, there is an increased chance that $Y_{1s}$ is a ‘random low’, which is deflationary and may lead to under‐estimation of the treatment effect.

Under the null hypothesis, $Y_{11}$, $Y_{12}$, and $Y_{2s}$ can be viewed (approximately) as standard normal random variables (mean 0, variance 1). The observed $-\log\left(\mathrm{HR}\right)$ is approximately $Y_{1s}/\sqrt{\mathrm{tI}}$ at Stage 1 and $Y_{2s}/\sqrt{\left(1-t\right)I}$ at Stage 2. If we combine data from two stages, the combined log‐rank statistic can be approximated with

$$Y_{s}\;=\;\sqrt{t}\;Y_{1s}\;+\;\sqrt{1-t}\;Y_{2s}$$

#### 2.3.1 Conditions for Combining Versus Discarding Stage 1 Data

We define Δ as the discrepancy between Stage 1 and Stage 2,

$$\Delta \;=\;\left[-\log\left({\mathrm{HR}}_{1}\right)\right]\;-\;\left[-\log\left({\mathrm{HR}}_{2}\right)\right]\;=\;\frac{Y_{2s}}{\sqrt{\left(1-t\right)I}}\;-\;\frac{Y_{1s}}{\sqrt{\mathrm{tI}}}$$

which may be assessed before deciding whether to discard Stage 1 data. With consistency characterized with a cutoff point of c, we consider the following 3 hypothesis testing strategies:

- *Conservative*: Combine Stage 1 and Stage 2 data in the analysis if Δ<c, else discard Stage 1. For example, when $c=\log\left(1.15\right)$, data in rows 1–3 of Table 1 will be combined but data in row 4 will not.
- *Aggressive*: Combine Stage 1 and Stage 2 data in the analysis if Δ>−c, else discard Stage 1. For example, when $c=\log\left(1.15\right)$, data in rows 2–4 of Table 1 will be combined but data in row 1 will not.
- *Neutral*: Combine Stage 1 and Stage 2 data in the analysis if ∣Δ∣<c, else discard Stage 1. For example, when $c=\log\left(1.15\right)$, data in rows 2–3 of Table 1 will be combined but data in rows 1 and 4 will not.

The primary analysis, with or without including the Stage 1 data, will be conducted at the adjusted alpha‐level (α*) to control the overall Type error at α. Note, the conservative strategy may be favored by regulatory agencies and the aggressive strategy may be preferred by trial sponsors. The neutral strategy strikes a balance between the two, offering a middle ground where the conservative approach may be seen as too restrictive by sponsors, while the aggressive approach could be perceived as cherry‐picking by regulators.

#### 2.3.2 Overall Type I Error Under the Neutral Strategy

For clarity, we only provide details for the *neutral* strategy. The other two have similar but simpler forms, which are provided in the Supporting Information.

Let A,B,C,D be shorthand for the principal events that yield a Type I error, partitioned by whether Stage 1 is “high” ($Y_{1s}=\max\left\{Y_{11},Y_{12}\right\}$) or “low” ($Y_{1s}=\min\left\{Y_{11},Y_{12}\right\}$), whether ∣Δ∣<c or ∣Δ∣>c, and whether the final test statistic exceeds the critical threshold $z_{1-\alpha *}$, the normal quantile at the 1−α* level. Below, each probability is also conditioned on whether a “winner” or “loser” is picked.

- A: The event that ∣Δ∣<c and $Y_{s}>z_{1-\alpha *}$ given $Y_{1s}=\max\left\{Y_{11},Y_{12}\right\}$.
- B: The event that ∣Δ∣<c and $Y_{s}>z_{1-\alpha *}$ given $Y_{1s}=\min\left\{Y_{11},Y_{12}\right\}$.
- C: The event that ∣Δ∣>c and $Y_{2s}>z_{1-\alpha *}$ given $Y_{1s}=\max\left\{Y_{11},Y_{12}\right\}$.
- D: The event that ∣Δ∣>c and $Y_{2s}>z_{1-\alpha *}$ given $Y_{1s}=\min\left\{Y_{11},Y_{12}\right\}$.

Hence, controlling the overall Type I error under the neutral strategy requires:

(1) $$\mathrm{Pr}\left(\text{Type}\;I\;\text{Error}\right)\;=\;w\;P\left(A\right)+\left(1-w\right)\;P\left(B\right)+w\;P\left(C\right)+\left(1-w\right)\;P\left(D\right)\;=\;\alpha$$

We solve Equation (1) to find the adjusted threshold α* to control Type I error rate at the level of α. Because these integrals rarely admit closed‐form solutions, we compute α* numerically. The R‐code of this calculation is provided in Appendix 1. Once α* is found, the final test is: If ∣Δ∣<c, combine Stage 1 and Stage 2 and compare $Y_{s}=\sqrt{t}\;Y_{1s}+\sqrt{1-t}\;Y_{2s}$ to $z_{1-\alpha *}$; otherwise, discard Stage 1 and compare $Y_{2s}$ to $z_{1-\alpha *}$. Note that when w<0.5 in practice, it will be set at 0.5 in the computation to ensure α*<α, thereby strongly controlling the Type I error [6].

Similar code can be written for *conservative* (Δ<c) or *aggressive* (Δ>−c) rules. In all cases, the approach ensures that the total probability of a false positive is controlled at the α level despite the possible inconsistency and imperfect dose selection.

#### 2.3.3 Derivation of Probability of Four Events *A*, *B*, *C*, *D*

The calculation of the probability of events A,B,C,D can be expanded in detail. For simplicity, let us outline A as an example:

$$\begin{array}{c} \mathrm{Pr}\left(A\right)\;=\;\mathrm{Pr}\left(|,\Delta ,|,=,\left|\frac{Y_{1s}}{\sqrt{\mathrm{tI}}},-,\frac{Y_{2s}}{\sqrt{\left(1-t\right)I}}\right|,<c,,,\;\sqrt{t}\;Y_{1s}+\sqrt{1-t}\;Y_{2s}>z_{\left(1-\alpha *\right)},\;|Y_{1s}=\max\left\{Y_{11},Y_{12}\right\}\right) \end{array}$$

By definition, $Y_{1s}=\max\left(Y_{11},Y_{12}\right)$ under the “picked winner” scenario. We rewrite $\mathrm{Pr}\left(\Delta <c\right)$ as two inequalities:

$$\mathrm{Pr}\left(\left(\frac{\max\left(Y_{11},Y_{12}\right)}{\sqrt{\mathrm{tI}}}-\frac{Y_{2s}}{\sqrt{\left(1-t\right)I}}\right)<c\right)=\mathrm{Pr}\left(\left(\frac{Y_{11}}{\sqrt{\mathrm{tI}}}-\frac{Y_{2s}}{\sqrt{\left(1-t\right)I}}\right)<c,\left(\frac{Y_{12}}{\sqrt{\mathrm{tI}}}-\frac{Y_{2s}}{\sqrt{\left(1-t\right)I}}\right)<c\right)$$

Using similar rule, we calculate $\mathrm{Pr}\left(A\right)$ as follows:

$$\begin{array}{c} \;\mathrm{Pr}\left(A\right)\\ =\mathrm{Pr}\left(\frac{\max\left\{Y_{11},Y_{12}\right\}}{\sqrt{\mathrm{tI}}}-\frac{Y_{2s}}{\sqrt{\left(1-t\right)I}}<c\right)-\mathrm{Pr}\left(\frac{\max\left\{Y_{11},Y_{12}\right\}}{\sqrt{\mathrm{tI}}}-\frac{Y_{2s}}{\sqrt{\left(1-t\right)I}}<-c\right)\\ \;-\;\mathrm{Pr}\left(\frac{\max\left\{Y_{11},Y_{12}\right\}}{\sqrt{\mathrm{tI}}}-\frac{Y_{2s}}{\sqrt{\left(1-t\right)I}}<c,\;\sqrt{t}\;\max\left\{Y_{11},Y_{12}\right\}+\sqrt{1-t}\;Y_{2s}<z_{\left(1-\alpha *\right)}\right)\\ \;+\;\mathrm{Pr}\left(\frac{\max\left\{Y_{11},Y_{12}\right\}}{\sqrt{\mathrm{tI}}}-\frac{Y_{2s}}{\sqrt{\left(1-t\right)I}}<-c,\;\sqrt{t}\;\max\left\{Y_{11},Y_{12}\right\}+\sqrt{1-t}\;Y_{2s}<z_{\left(1-\alpha *\right)}\right)\\ =\mathrm{Pr}\left(\frac{Y_{11}}{\sqrt{\mathrm{tI}}}-\frac{Y_{2s}}{\sqrt{\left(1-t\right)I}}<c,\frac{Y_{12}}{\sqrt{\mathrm{tI}}}-\frac{Y_{2s}}{\sqrt{\left(1-t\right)I}}<c\right)\\ \;-\mathrm{Pr}\left(\frac{Y_{11}}{\sqrt{\mathrm{tI}}}-\frac{Y_{2s}}{\sqrt{\left(1-t\right)I}}<-c,\frac{Y_{12}}{\sqrt{\mathrm{tI}}}-\frac{Y_{2s}}{\sqrt{\left(1-t\right)I}}<-c\right)\\ \;-\mathrm{Pr}\left(\frac{Y_{11}}{\sqrt{\mathrm{tI}}}-\frac{Y_{2s}}{\sqrt{\left(1-t\right)I}}<c,\frac{Y_{12}}{\sqrt{\mathrm{tI}}}-\frac{Y_{2s}}{\sqrt{\left(1-t\right)I}}<c,\sqrt{t}Y_{11}+\sqrt{1-t}Y_{2s}<z_{1-\alpha *},\sqrt{t}Y_{12}+\sqrt{1-t}Y_{2s}<z_{1-\alpha *}\;\right)\\ \;+\mathrm{Pr}\left(\frac{Y_{11}}{\sqrt{\mathrm{tI}}}-\frac{Y_{2s}}{\sqrt{\left(1-t\right)I}}<-c,\frac{Y_{12}}{\sqrt{\mathrm{tI}}}-\frac{Y_{2s}}{\sqrt{\left(1-t\right)I}}<-c,\sqrt{t}Y_{11}+\sqrt{1-t}Y_{2s}<z_{1-\alpha *},\sqrt{t}Y_{12}+\sqrt{1-t}Y_{2s}<z_{1-\alpha *}\;\right) \end{array}$$

Similarly, we have

$$\begin{array}{c} P\left(B\right)=P\left(\frac{Y_{11}}{\sqrt{\mathrm{tI}}}-\frac{Y_{2s}}{\sqrt{\left(1-t\right)I}}>-c,\frac{Y_{12}}{\sqrt{\mathrm{tI}}}-\frac{Y_{2s}}{\sqrt{\left(1-t\right)I}}>-c,\sqrt{t}Y_{11}+\sqrt{1-t}Y_{2s}>z_{1-\alpha *},\sqrt{t}Y_{12}+\sqrt{1-t}Y_{2s}>z_{1-\alpha *}\;\right)\\ \;-\;P\left(\frac{Y_{11}}{\sqrt{\mathrm{tI}}}-\frac{Y_{2s}}{\sqrt{\left(1-t\right)I}}>c,\frac{Y_{12}}{\sqrt{\mathrm{tI}}}-\frac{Y_{2s}}{\sqrt{\left(1-t\right)I}}>c,\sqrt{t}Y_{11}+\sqrt{1-t}Y_{2s}>z_{1-\alpha *},\sqrt{t}Y_{12}+\sqrt{1-t}Y_{2s}>z_{1-\alpha *}\;\right)\\ P\left(C\right)=P\left(\frac{Y_{11}}{\sqrt{\mathrm{tI}}}-\frac{Y_{2s}}{\sqrt{\left(1-t\right)I}}<-c,\frac{Y_{12}}{\sqrt{\mathrm{tI}}}-\frac{Y_{2s}}{\sqrt{\left(1-t\right)I}}<-c,Y_{2s}>z_{1-\alpha *}\;\right)+P\left(Y_{2s}>z_{1-\alpha *}\right)\\ \;-P\left(\frac{Y_{11}}{\sqrt{\mathrm{tI}}}-\frac{Y_{2s}}{\sqrt{\left(1-t\right)I}}<c,\frac{Y_{12}}{\sqrt{\mathrm{tI}}}-\frac{Y_{2s}}{\sqrt{\left(1-t\right)I}}<c,Y_{2s}>z_{1-\alpha *}\;\right)\\ =P\left(\frac{Y_{11}}{\sqrt{\mathrm{tI}}}-\frac{Y_{2s}}{\sqrt{\left(1-t\right)I}}<-c,\frac{Y_{12}}{\sqrt{\mathrm{tI}}}-\frac{Y_{2s}}{\sqrt{\left(1-t\right)I}}<-c,Y_{2s}>z_{1-\alpha *}\;\right)+\alpha *\\ \;-P\left(\frac{Y_{11}}{\sqrt{\mathrm{tI}}}-\frac{Y_{2s}}{\sqrt{\left(1-t\right)I}}<c,\frac{Y_{12}}{\sqrt{\mathrm{tI}}}-\frac{Y_{2s}}{\sqrt{\left(1-t\right)I}}<c,Y_{2s}>z_{1-\alpha *}\;\right)\\ P\left(D\right)=P\left(\frac{Y_{11}}{\sqrt{\mathrm{tI}}}-\frac{Y_{2s}}{\sqrt{\left(1-t\right)I}}>c,\frac{Y_{12}}{\sqrt{\mathrm{tI}}}-\frac{Y_{2s}}{\sqrt{\left(1-t\right)I}}>c,Y_{2s}>z_{1-\alpha *}\;\right)+\alpha *\\ \;-P\left(\frac{Y_{11}}{\sqrt{\mathrm{tI}}}-\frac{Y_{2s}}{\sqrt{\left(1-t\right)I}}>-c,\frac{Y_{12}}{\sqrt{\mathrm{tI}}}-\frac{Y_{2s}}{\sqrt{\left(1-t\right)I}}>-c,Y_{2s}>z_{1-\alpha *}\;\right) \end{array}$$

*In summary*, under the neutral strategy, we pay an extra Type I error penalty if we frequently combine Stage 1 data that are possibly a random high (when w>0.5). Numerically, we find α* with the code in the Appendix 1. The same logic applies for the conservative or aggressive strategies, with simpler conditions on Δ.

## 3 Numerical Results

**FIGURE 1:**
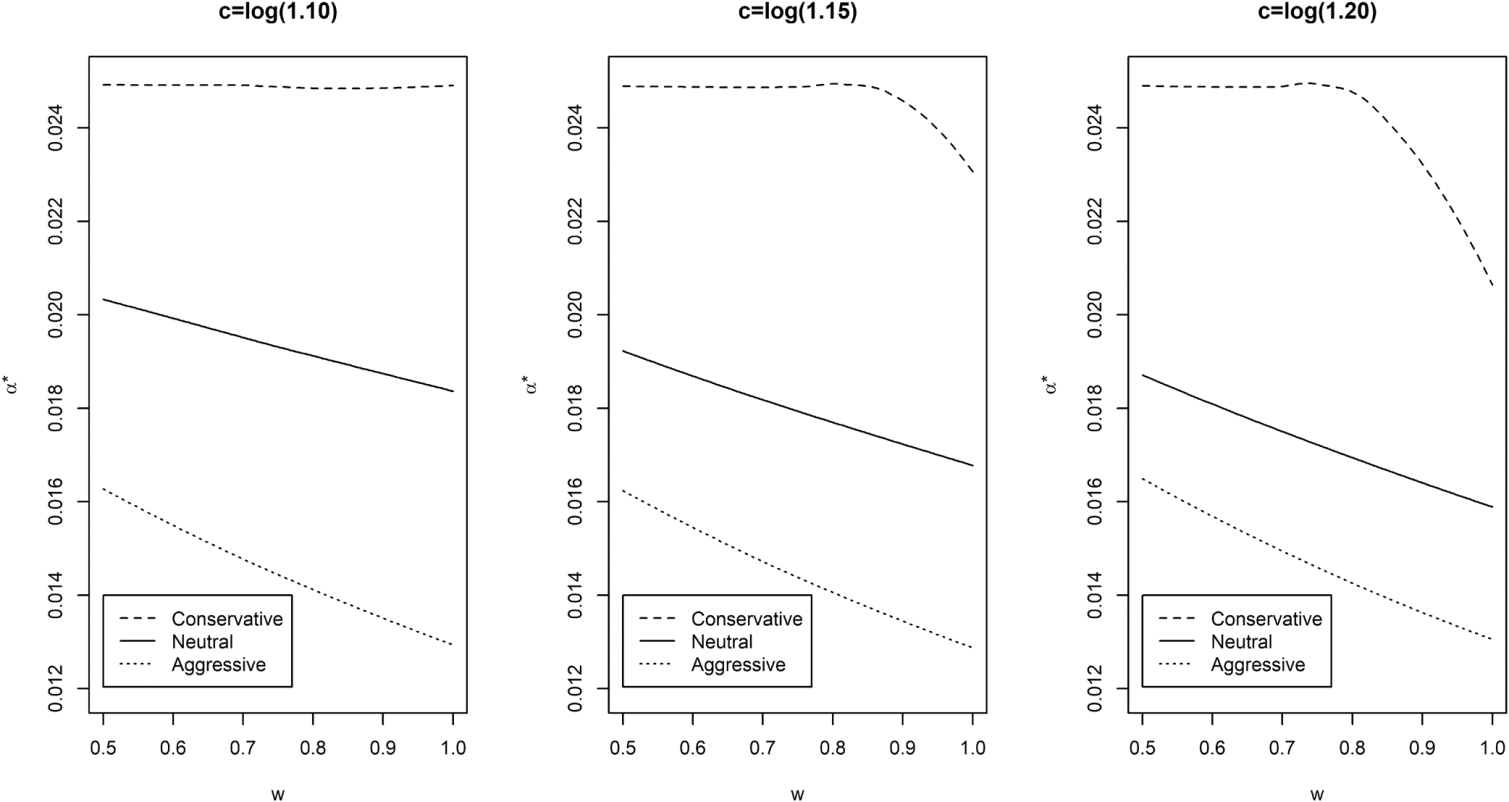
Plot of α* as w increases from 0.5 to 1.0 for the three statistical testing strategies when $c=\log\left(1.10\right)$, $\log\left(1.15\right)$, or $\log\left(1.20\right)$.

In a typical adaptive Phase 2/3 design with dose selection, about 20% of the patients may be from Phase 2. Since they have longer follow‐up than Phase 3 patients, their contribution to the pooled analysis is greated (e.g., about 30%). Figure 1 shows α* values across various scenarios when t is set at 30% as an example when w ranges from 0.5 to 1. Clearly, little penalty needs to be paid under the conservative strategy, while a lot must be paid under the aggressive strategy. The penalty for the neutral strategy falls in between. As expected, the penalty increases with c and w. Under the neutral strategy, α* decreases slowly from 0.0204 at w=0.5 to 0.0183 at w=1.0 when $c=\log\left(1.10\right)$ and from 0.0188 at w=0.5 to 0.0160 at w=1.0 when $c=\log\left(1.20\right)$ with the numbers fall in between when $c=\log\left(1.15\right)$.

**FIGURE 2:**
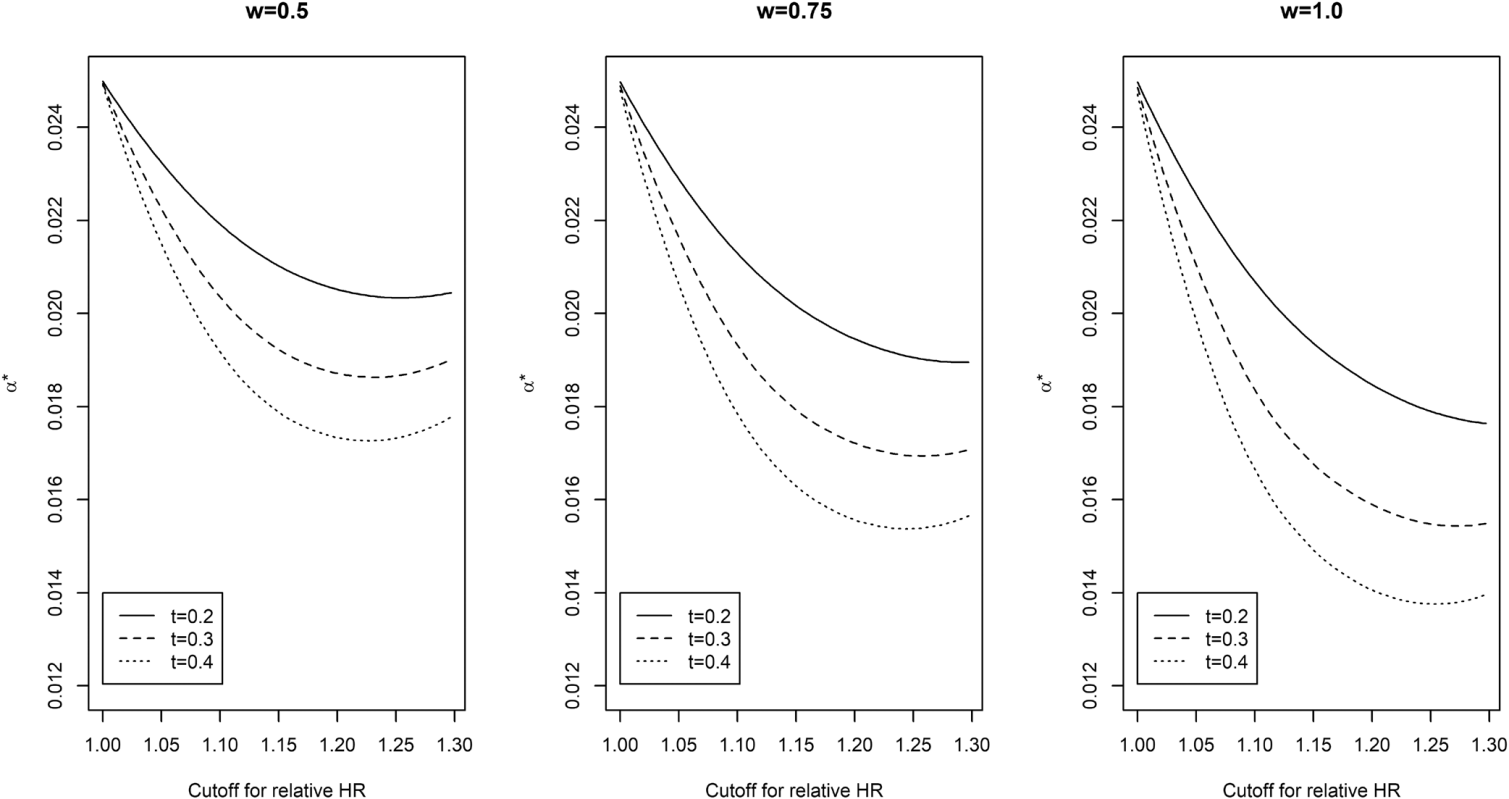
Plot of α* under the neutral strategy as c increases from $\log\left(1.0\right)$ to $\log\left(1.3\right)$ when t=0.2, 0.3, or 0.4.

Figure 2 takes a closer look at α* under the neutral strategy when w is set at an extreme value of special interest (0.5 or 1.0) as well as at the intermediate point of 0.75. While α* decreases as t increases, as expected, it is not monotonic in c and remains relative stable when c is in a range of practical interest (e.g., $\log\left(1.1\right)$ to $\log\left(1.2\right)$). In practice, patients treated at the unselected doses may have a different survival follow‐up and treatment pattern compared to those treated at the selected dose, it is hard to know whether a true winner or loser has been picked based on the observed survival data. When the survival data is reliable, w would be 1 if a winner is picked and 0 if a loser is picked (0 is technically changed to 0.5 in α* calculation to maintain strong control of Type I error). While a conventional *p* value combination test may be suitable for w=1, no penalty is needed for w=0 [7]. When the survival data is unreliable, we would need to rely on an estimated w to calculate α* which is feasible if the dose selection criteria are binding [6]. Otherwise, it is reasonable to set it at 0.5 if the overall efficacy and safety data are comparable between the two doses. In this case, an upper bound for w based on data maturity at dose selection may be considered for a sensitivity analysis (Appendix 2).

## 4 Discussion and Conclusion

Our framework incorporates both consistency between the two stages and imperfection in dose‐selection into the primary analysis of an adaptive Phase 2/3 design with dose optimization. Sponsors and regulators can jointly decide on the cutoff for consistency measure and a testing strategy (conservative, neutral, or aggressive) that reflect their tolerance for inconsistency. While the conservative strategy may be preferred by the regulatory agencies, sponsors only need to pay minimal penalty; While the aggressive strategy may be preferred by sponsors, substantial penalty must be paid. The neutral strategy represents a reasonable compromise. Choice of the cutoff point for measuring consistency not only impacts α* but also study power, a topic under investigation. The introduction of c under the neutral strategy results in a hybrid design: it becomes more inferential as c increases and is more operational as c decreases. To what extent the adaptive design is inferential or operational is data‐driven and guided by c. In practice, a reasonable starting point for c may be between $\log\left(1.1\right)$ and $\log\left(1.2\right)$ under a typical adaptive Phase 2/3 design setting in oncology drug development.

It is increasingly realized that the assumption of w=1 under the prevailing statistical approaches is too conservative in contemporary oncology drug development. While novel alternative approaches have been proposed [7, 8, 9] with some aiming to essentially relax the assumption, none have addressed the inconsistency concern. With the inconsistency issue exacerbated by imperfect dose selection, this investigation paves the way for further research in a less explored area.

We have provided a reasonable bound for w in this paper and illustrated its estimation elsewhere. Proper bounding or estimation of w is crucial, as is choosing c in a manner consistent with clinical expectations of how large stage‐to‐stage differences might be. Further research may address power considerations and ways to optimize c or w based on pilot data or prior knowledge of endpoint correlations.

In conclusion, we have presented a statistical method to manage consistency concerns in adaptive Phase 2/3 designs with dose optimization. By specifying an inconsistency cutoff (c), incorporating the true probability of picking‐the‐winner (w), and controlling Type I error at an adjusted α*, sponsors and regulators can systematically decide whether—and how—to pool Stage 1 and Stage 2 data. This approach can be tailored to meet varying degrees of regulatory conservatism or sponsor objectives.

## Conflicts of Interest

The authors may benefit from the publication of this work.

## Supporting information


**Data S1:** pst70031‐sup‐0001‐Supinfo.pdf.

## Data Availability

Data sharing is not applicable to this article as no datasets were generated or analysed during the current study.

## Appendix 1 R Code for Calculation of α* for Overall Type I Error Control

Below is sample R code for the neutral strategy. Similar code can be written for the conservative or aggressive strategies by adjusting the logic for Δ.library(mvtnorm)eq1 <‐ function(c, t, I, astar) {  s1 <‐ matrix(c(1/(t*I)+1/((1‐t)*I), 1/(2*t*I)+1/((1‐t)*I), 1/(2*t*I)+1/((1‐t)*I), 1/(t*I)+1/((1‐t)*I)), 2, 2)  s2a <‐ s1  s2b <‐ matrix(c(0, ‐1/(2*sqrt(I)), ‐1/(2*sqrt(I)), 0), 2, 2)  s2c <‐ matrix(c(1, 1‐t/2, 1‐t/2, 1), 2, 2)  s2 <‐ rbind(cbind(s2a, s2b), cbind(s2b, s2c))  f11 <‐ pmvnorm(upper = c(c, c), sigma = s1)[1]  f12 <‐ pmvnorm(upper = c(‐c, ‐c), sigma = s1)[1]  f13 <‐ pmvnorm(upper = c(c, c, qnorm(1‐astar), qnorm(1‐astar)), sigma = s2)[1]  f14 <‐ pmvnorm(upper = c(‐c, ‐c, qnorm(1‐astar), qnorm(1‐astar)), sigma = s2)[1]  f11 ‐ f12 ‐ f13 + f14}eq2 <‐ function(c, t, I, astar) {  s1 <‐ matrix(c(1/(t*I)+1/((1‐t)*I), 1/(2*t*I)+1/((1‐t)*I), 1/(2*t*I)+1/((1‐t)*I), 1/(t*I)+1/((1‐t)*I)), 2, 2)  s2a <‐ s1  s2b <‐ matrix(c(0, ‐1/(2*sqrt(I)), ‐1/(2*sqrt(I)), 0), 2, 2)  s2c <‐ matrix(c(1, 1‐t/2, 1‐t/2, 1), 2, 2)  s2 <‐ rbind(cbind(s2a, s2b), cbind(s2b, s2c))  f21 <‐ pmvnorm(lower = c(‐c, ‐c, qnorm(1‐astar), qnorm(1‐astar)), sigma = s2)[1]  f22 <‐ pmvnorm(lower = c(c, c, qnorm(1‐astar), qnorm(1‐astar)), sigma = s2)[1]  f21 ‐ f22}eq3 <‐ function(c, t, I, astar) {  s1 <‐ matrix(c(1/(t*I)+1/((1‐t)*I), 1/(2*t*I)+1/((1‐t)*I), 1/(2*t*I)+1/((1‐t)*I), 1/(t*I)+1/((1‐t)*I)), 2, 2)  s3 <‐ rbind(cbind(s1, rep(‐1/sqrt((1‐t)*I), 2)), c(rep(‐1/sqrt((1‐t)*I), 2), 1))  f31 <‐ astar  f32 <‐ pmvnorm(upper = c(‐c, ‐c, Inf),           lower = c(‐Inf, ‐Inf, qnorm(1‐astar)),           sigma = s3)[1]  f33 <‐ pmvnorm(upper = c(c, c, Inf),           lower = c(‐Inf, ‐Inf, qnorm(1‐astar)),           sigma = s3)[1]  f31 + f32 ‐ f33}eq4 <‐ function(c, t, I, astar) {  s1 <‐ matrix(c(1/(t*I)+1/((1‐t)*I), 1/(2*t*I)+1/((1‐t)*I), 1/(2*t*I)+1/((1‐t)*I), 1/(t*I)+1/((1‐t)*I)), 2, 2)  s3 <‐ rbind(cbind(s1, rep(‐1/sqrt((1‐t)*I), 2)), c(rep(‐1/sqrt((1‐t)*I), 2), 1))  f41 <‐ astar  f42 <‐ pmvnorm(lower = c(c, c, qnorm(1‐astar)), sigma = s3)[1]  f43 <‐ pmvnorm(lower = c(‐c, ‐c, qnorm(1‐astar)), sigma = s3)[1]  f41 + f42 ‐ f43}calc_typeI <‐ function(astar, c, t, I, w) {  eq1(c, t, I, astar)*w +    eq2(c, t, I, astar)*(1‐w) +    eq3(c, t, I, astar)*w +    eq4(c, t, I, astar)*(1‐w)}solve_astar <‐ function(alpha, c, t, I, w) {  op_fn <‐ function(astar) {    (calc_typeI(astar, c, t, I, w) ‐ alpha)^2  }  optimize(op_fn, lower=0, upper=alpha)$minimum}# Example usage:# solve_astar(alpha=0.025, c=log(1.1), t=0.3, I=510/4, w=0.6)

## Appendix 2 Upper Bound of w

With a slight absues of notations, let N represent the projected number of events for Stage 1 patients concerning the Phase 3 primary endpoint at the final analysis, and n be the number of their events at the dose selection. The data maturity for the primary endpoint (information fraction) at dose selection is s=n/N. When the survival data for the unselected dose is unreliable, s can be estimated using data from the selected dose and the control.

With a slight absues of notations again, let X be the log‐rank test statistic between the two doses at dose selection and Y be the log‐rank test statistic between the two doses at the final analysis. By noticing that X>0 achieves the same goal of picking‐the‐winner as in the main text, the probability that a superior dose at dose selection is also superior at the final analysis is approximately $w=\mathrm{Pr}\left(Y>0|X>0\right)=\mathrm{Pr}\left(Y>0,X>0\right)/\mathrm{Pr}\left(X>0\right)=2\mathrm{Pr}\left(Y>0,X>0\right)$. Under the null hypothesis, in the ideal world when the treatment effect at dose selection is the same as at the final analysis, the two standard normal random variables have an approximate correlation of $\sqrt{s}$. The calculation of w is straightforward once s is available. For example, in a study when the final analysis is conducted after 75% of the patients have an event, s would be 20% if 15% of them have an event at the dose selection (i.e., 0.15/0.75=0.20), and in this case w=0.65.

In reality, the treatment effects may be different and we have to take a suboptimal approach of using early efficacy and safety data for dose selection. Consequently, the actual w can only be smaller.
