# Supplementary material for: Adjustment for Inconsistency in Adaptive Phase 2/3 Designs With Dose Optimization
Source: Pharm Stat. 2025 Sep 26;24(6):e70031. doi: 10.1002/pst.70031 (PMC12467493; doi:10.1002/pst.70031)
Supplement: Supplementary file 1 — Data S1: pst70031‐sup‐0001‐Supinfo.pdf. [file PST-24-0-s001.pdf]

Supplementary Material: Adjustment for Inconsistency in Adaptive Phase 2/3 Designs with Dose Optimization - Conservative and Aggressive Strategies

For each strategy, we partition false-positive events into A, B, C, D exactly as in Section 2.3.2. The overall Type I error is constrained by

$$w\big[P(A) + P(C)\big] + (1-w)\big[P(B) + P(D)\big] = \alpha. \tag{$*$}$$

The probabilities of these events are provided below.

For brevity, we define the following two quantities:

$$\delta_1 = \frac{Y_{11}}{\sqrt{tI}} - \frac{Y_{2s}}{\sqrt{(1-t)I}}, \qquad \delta_2 = \frac{Y_{12}}{\sqrt{tI}} - \frac{Y_{2s}}{\sqrt{(1-t)I}}.$$

1. Conservative Strategy $\big($Combine if $\boldsymbol{\Delta < c}\big)$

Events.

$$\begin{aligned}
A_{\text{cons}} &: \{\Delta < c,\ Y_{1s+2s} > z_{1-\alpha^*}\} \cap \{Y_{1s} = \max\{Y_{1s}, Y_{2s}\}\},\\
B_{\text{cons}} &: \{\Delta < c,\ Y_{1s+2s} > z_{1-\alpha^*}\} \cap \{Y_{1s} = \min\{Y_{1s}, Y_{2s}\}\},\\
C_{\text{cons}} &: \{\Delta \ge c,\ Y_{2s} > z_{1-\alpha^*}\} \cap \{Y_{1s} = \max\{Y_{1s}, Y_{2s}\}\},\\
D_{\text{cons}} &: \{\Delta \ge c,\ Y_{2s} > z_{1-\alpha^*}\} \cap \{Y_{1s} = \min\{Y_{1s}, Y_{2s}\}\}.
\end{aligned}$$

For the *winner* arm ($Y_{1s} = \max\{Y_{1s}, Y_{2s}\}$), we have

$$\begin{aligned}
\Delta < c &\iff \delta_1 < c \text{ and } \delta_2 < c\\
\Delta \ge c &\iff \delta_1 \ge c \text{ or } \delta_2 \ge c
\end{aligned}$$

Hence:

$$P(A_{\text{cons}}) = \Pr(\delta_1 < c,\ \delta_2 < c,\ Y_{1s+2s} > z_{1-\alpha^*}), \tag{C1}$$
$$P(C_{\text{cons}}) = \alpha^* - \Pr(\delta_1 < c,\ \delta_2 < c,\ Y_{2s} > z_{1-\alpha^*}). \tag{C2}$$

For the *loser* arm ($Y_{1s} = \min\{Y_{1s}, Y_{2s}\}$), we have

$$\begin{aligned}
\Delta < c &\iff \delta_1 < c \text{ or } \delta_2 < c\\
\Delta \ge c &\iff \delta_1 \ge c \text{ and } \delta_2 \ge c
\end{aligned}$$

Using the complement:

$$P(B_{\text{cons}}) = \alpha^* - \Pr(\delta_1 \ge c,\ \delta_2 \ge c,\ Y_{1s+2s} > z_{1-\alpha^*}), \tag{C3}$$
$$P(D_{\text{cons}}) = \Pr(\delta_1 \ge c,\ \delta_2 \ge c,\ Y_{2s} > z_{1-\alpha^*}). \tag{C4}$$

Substituting (C1)–(C4) into ($*$) and solving numerically yields the adjusted level α^*_{cons}.

2. Aggressive Strategy $\big($Combine if $\boldsymbol{\Delta > -c}\big)$

Events.

$$
\begin{aligned}
A_{\text{agg}} &: \{\Delta > -c,\ Y_{1s+2s} > z_{1-\alpha^*}\} \cap \{Y_{1s} = \max\{Y_{1s}, Y_{2s}\}\},\\
B_{\text{agg}} &: \{\Delta > -c,\ Y_{1s+2s} > z_{1-\alpha^*}\} \cap \{Y_{1s} = \min\{Y_{1s}, Y_{2s}\}\},\\
C_{\text{agg}} &: \{\Delta \le -c,\ Y_{2s} > z_{1-\alpha^*}\} \cap \{Y_{1s} = \max\{Y_{1s}, Y_{2s}\}\},\\
D_{\text{agg}} &: \{\Delta \le -c,\ Y_{2s} > z_{1-\alpha^*}\} \cap \{Y_{1s} = \min\{Y_{1s}, Y_{2s}\}\}.
\end{aligned}
$$

For the *winner* arm ($Y_{1s} = \max\{Y_{1s}, Y_{2s}\}$), we have

$$
\begin{aligned}
\Delta \le -c &\iff \delta_1 \le -c \text{ and } \delta_2 \le -c\\
\Delta > -c &\iff \delta_1 > -c \text{ or } \delta_2 > -c
\end{aligned}
$$

Hence:

$$
\begin{aligned}
P(A_{\text{agg}}) &= \alpha^* - \Pr(\delta_1 \le -c,\ \delta_2 \le -c,\ Y_{1s+2s} > z_{1-\alpha^*}), && \text{(A1)}\\
P(C_{\text{agg}}) &= \Pr(\delta_1 \le -c,\ \delta_2 \le -c,\ Y_{2s} > z_{1-\alpha^*}). && \text{(A2)}
\end{aligned}
$$

For the *loser* arm:

$$
\begin{aligned}
\Delta \le -c &\iff \delta_1 \le -c \text{ or } \delta_2 \le -c\\
\Delta > -c &\iff \delta_1 > -c \text{ and } \delta_2 > -c
\end{aligned}
$$

Hence:

$$
\begin{aligned}
P(B_{\text{agg}}) &= \Pr(\delta_1 > -c,\ \delta_2 > -c,\ Y_{1s+2s} > z_{1-\alpha^*}), && \text{(A3)}\\
P(D_{\text{agg}}) &= \alpha^* - \Pr(\delta_1 > -c,\ \delta_2 > -c,\ Y_{2s} > z_{1-\alpha^*}). && \text{(A4)}
\end{aligned}
$$

Insert (A1)–(A4) into $(*)$ to compute α^*_{agg}.
